# Phage-Based Biosanitation Strategies for Minimizing Persistent *Salmonella* and *Campylobacter* Bacteria in Poultry

**DOI:** 10.3390/ani13243826

**Published:** 2023-12-12

**Authors:** Jaume Jordá, Laura Lorenzo-Rebenaque, Laura Montoro-Dasi, Ana Marco-Fuertes, Santiago Vega, Clara Marin

**Affiliations:** 1 Departamento de Producción y Sanidad Animal, Salud Pública Veterinaria y Ciencia y Tecnología de los Alimentos, Facultad de Veterinaria, Instituto de Ciencias Biomédicas, Universidad Cardenal Herrera-CEU, CEU Universities, Calle Santiago Ramón y Cajal 20, 46115 Alfara del Patriarca, Spain; jaume.jorda@uchceu.es (J.J.); laura.montoro@uchceu.es (L.M.-D.); ana.marcofuertes@uchceu.es (A.M.-F.); svega@uchceu.es (S.V.); 2 Institute of Animal Science and Technology, Universitat Politècnica de València, 46022 Valencia, Spain; laulore@upv.es

**Keywords:** bacteriophages, *Salmonella*, *Campylobacter*, persistent clones, poultry

## Abstract

**Simple Summary:**

Implementing strategies to reduce harmful bacteria in food animal production plays a vital role in ensuring safer food for consumers. The food industry, especially in poultry and swine farming, faces significant challenges, including antibiotic-resistant and disinfection-resistant zoonotic bacteria. In this context, bacteriophages, which are viruses that attack bacteria, have emerged as a promising tool to control these bacteria throughout the food production process, from the animals and farms to the end product. Bacteriophages offer several advantages as a biocontrol agent, including their precision in targeting specific bacteria, ability to replicate, adaptability, low risk of toxicity and ease of isolation. Developing them as a biocontrol tool is particularly appealing because it aligns with the use of environmentally friendly “green” technology to combat harmful bacteria. This review provides a comprehensive overview of how phage-based strategies can help reduce persistent *Salmonella* and *Campylobacter* bacteria in poultry, contributing to safer food production.

**Abstract:**

Control strategies to minimize pathogenic bacteria in food animal production are one of the key components in ensuring safer food for consumers. The most significant challenges confronting the food industry, particularly in the major poultry and swine sectors, are antibiotic resistance and resistance to cleaning and disinfection in zoonotic bacteria. In this context, bacteriophages have emerged as a promising tool for zoonotic bacteria control in the food industry, from animals and farm facilities to the final product. Phages are viruses that infect bacteria, with several advantages as a biocontrol agent such as high specificity, self-replication, self-limitation, continuous adaptation, low inherent toxicity and easy isolation. Their development as a biocontrol agent is of particular interest, as it would allow the application of a promising and even necessary “green” technology to combat pathogenic bacteria in the environment. However, bacteriophage applications have limitations, including selecting appropriate phages, legal restrictions, purification, dosage determination and bacterial resistance. Overcoming these limitations is crucial to enhance phage therapy’s effectiveness against zoonotic bacteria in poultry. Thus, this review aims to provide a comprehensive view of the phage-biosanitation strategies for minimizing persistent *Salmonella* and *Campylobacter* bacteria in poultry.

## 1. Introduction

*Salmonella* and *Campylobacter* constitute the most common zoonotic pathogens involved in human outbreaks of foodborne disease in the developed world, followed by other important pathogens such as *Escherichia coli*, *Listeria* and *Vibrio cholerae* [1,2]. Even though many recent outbreaks have been linked to pork meat, fresh products and fruit, human clinical cases and outbreaks are still often associated with and attributed to poultry meat and eggs [3,4].

Since the mid-20th century, *Salmonella* has been acknowledged as a zoonotic pathogen with a mass impact on public health worldwide [5,6]. In Europe, a total of 773 salmonellosis foodborne outbreaks were reported across 27 European countries in 2021, resulting in 6755 cases, 1123 hospitalizations and 1 death [1]. Moreover, it is assumed that only 1 in 7 cases is diagnosed [7,8,9], since up to 80% of salmonellosis cases are not associated with a known outbreak; they are indeed considered sporadic illnesses, and the majority go undiagnosed [10]. Although different serotypes have been associated with salmonellosis, recent studies have reported *Salmonella enterica* subsp. *enterica* serovar Infantis as being widely spread through chicken meat [1,11] and *Salmonella enterica* subsp. *enterica* serovar Enteritidis through eggs [1,12]. Epidemiological surveillance suggests that the majority of outbreaks are associated with antibiotic-resistant bacteria, leading to increased severity of systemic disease, treatment failures and a surge in hospitalizations [10].

Public health implications of *Campylobacter* infections have evolved over more than a century, and currently, they are one of the major causes of foodborne illness worldwide [13,14]. In Europe, a total of 249 campylobacteriosis foodborne outbreaks were reported across 27 European countries in 2021, resulting in 1051 cases, 134 hospitalizations and 6 deaths [1]. Poultry, especially chicken meat, has been linked to 50 to 80% of human cases [15]. The role of poultry in the epidemiology of human disease was proven in Belgium in 1999, during the dioxin crisis when high levels of dioxins in chicken feed produced a reduction in human poultry meat consumption, resulting in a 40% reduction in human cases of campylobacteriosis [16]. Since then, poultry has been considered the natural reservoir of *Campylobacter jejuni* [17]. 

Both bacteria can colonize the poultry gut, especially the caeca, at high levels without exhibiting symptoms or a loss in production parameters, potentially leading to contamination of poultry meat during slaughter (in the case of *Salmonella* and *Campylobacter*) and the eggs (in the case of *Salmonella*), subsequently leading to human infections [4,17,18,19]. To control this situation, *Salmonella* National Control Programmes (SNCP) is aimed at on-farm *Salmonella* control (Directive 2003/2160/EC), and the process hygiene criterion for *Campylobacter* focused on slaughterhouse-level *Campylobacter* control (Regulation 2017/1495/EC) in each European Member State (MS). In this farm-to-table conceptualization, biosecurity measures, vaccination, alternative antibiotic products, cleaning and disinfection are the key tools to control these bacteria from the field [15,20]. Although strict measures have been proposed and regulations have improved, both bacteria continue to persist in the poultry industry [21]. This situation is closely related to the emergence of stress-tolerant and biofilm-forming *Salmonella* and *Campylobacter* which enhance their capacity to survive rigorous cleaning and disinfection protocols, facilitating their persistence in the facilities’ environment, even for more than one year [21,22]. In this context, cleaning and disinfection play a crucial role in eliminating bacteria. However, new sanitation techniques must be continually developed in order to control the emerging resistant pathogens and their impact on a global scale of any food safety program [23].

Currently, there is growing societal awareness and concern for environmental conservation, with an increasing intolerance towards the use of products that may have a detrimental impact on the environment and the surrounding ecosystem. In this regard, bacteriophages (or phages) represent an emerging up-and-coming “green” technology that could improve food safety with the potential to act against persistent *Salmonella* and *Campylobacter* [24]. The application of phages as a biocontrol tool emerges as a promising approach, not only as an animal treatment alternative to antibiotics, which has been undergoing an extensive body of work [25,26,27,28,29,30,31,32], but also as a complementary cleaning and disinfection tool for persistent strains, where they have been less studied.

## 2. General Considerations about Phages

Bacteriophages are ubiquitous in nature. They are a group of viruses whose life cycle is strictly associated with the bacterial cell. They are often called bacterial parasites, as they lack the cellular structure and enzyme systems needed to survive on their own. These incomplete organisms can only replicate inside the bacteria host [33,34]. Bacteriophages are specifically associated with a particular bacterial strain and exhibit strong bactericidal activity against Gram-positive and Gram-negative bacteria. Some phages show a specific affinity for individual types of bacteria, while others have a wide range of activity. Their specificity and range of activity are determined by the presence of receptors located on the bacteria’s surface, among which we can distinguish fragments of lipopolysaccharide (LPS), fimbriae and other surface proteins [35,36]. In this sense, monovalent phages target a single bacterial species, while polyvalent phages can attack two or more bacterial species. Studies indicate that phages that attack Gram-positive bacteria typically exhibit reduced efficacy against Gram-negative bacteria. Phages encode endolysins, hydrolases engaged in the lysis of the bacterial cell wall during the lytic cycle [37]. Endolysins can also destroy the peptidoglycan layer externally, making them effective antimicrobial agents, especially against Gram-positive bacteria. Nevertheless, the lysis of Gram-negative bacteria poses greater challenges due to their outer membrane, which impedes the access of endolysins to peptidoglycan. Lysis can be achieved by using permeabilizing agents [38,39,40,41].

**Figure 1:**
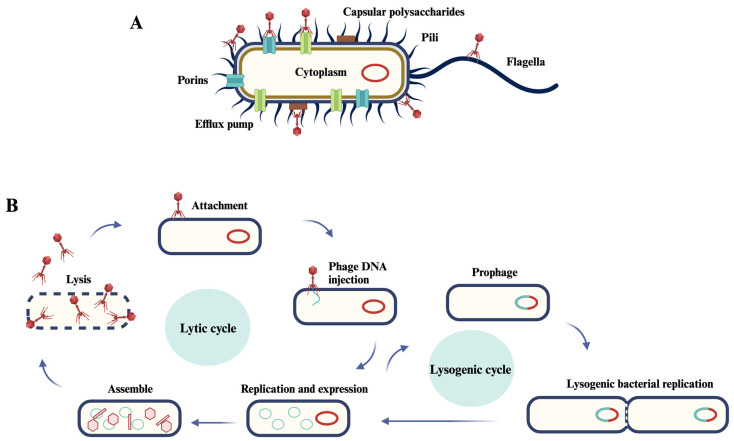
Mechanism of phage infection in bacteria. (**A**) Receptors for phage adsorption on bacteria. The color scheme distinguishes different components: blue represents porins, green represents efflux pumps, dark blue represents flagella and pili, and brown represents capsular polysaccharide. (**B**) Description of the phases of a phage lytic cycle and lysogenic cycle. The life cycle initiates with the attachment of the bacteriophage to receptors on the host bacteria’s cell membrane, followed by the injection of its genetic material. Subsequent stages include genome replication within the host cell, assembly of new phage progeny and their release. In contrast, the lysogenic cycle entails phage attachment to the host bacteria’s cell membrane receptors, genome injection, integration into the bacterial genome and replication alongside the host cell. Adapted from Huang et al. [45].

Phages can be divided into lytic phages and temperate phages, which have different life cycles, as shown in Figure 1. Lytic activity is typical of virulent phages, while lysogenic activity involves the integration of the bacteriophage genetic material with the bacterial chromosome and its replication as part of the bacteria DNA, leading to the appearance of a prophage [42]. The lytic cycle begins after the integration of the genetic material in the cell [43]. Once inside the cell, the phage’s genetic material replicates and assembles into new viral particles. To do this, during the eclipse period, the host’s biosynthetic machinery controls the synthesis of coating proteins, such as lysis proteins that cause lysis of the host cell [44]. The genetic material is packaged into the capsid, and the tail is attached to the head to form mature virions. When the number of viral particles in the bacterial cell reaches a critical level, the lysis process is triggered, which involves the rupture of the cell membrane of the bacterium and the release of the new viral particles into the environment, releasing new phages into the medium to begin another cycle of infection [43]. 

Conversely, lysogenic or temperate phages have the ability to exploit the host and deposit their genetic material within the infected bacterium for multiple generations [46,47,48]. In the lysogenic cycle, phage DNA replicates along with bacterial DNA, thus establishing a stable relationship. Usually in this cycle, the viral DNA is integrated into the bacterial chromosomal DNA of the host. In later stages during this life cycle, the change from lysogen to lytic form occurs, leading to activation of the lytic life cycle [49,50]. Lysogenic bacteriophages are typically not utilized directly as treatments because they have the ability to transfer genetic material from one bacterial cell to another, a phenomenon known as transduction. They can also transmit genes that enhance the virulence of the host, a process referred to as lysogenic conversion. Due to their replication cycle, they do not eliminate all the bacteria they infect, and a cell that carries a prophage within its genetic material becomes resistant to infection by it, a process known as immunity to superinfection. In contrast to lysogenic phages, lytic or virulent phages can multiply exponentially in bacterial culture and swiftly eradicate bacteria, irrespective of their resistance to antibiotics. 

## 3. Current Utilities and Legislations in the Use of Phage in Poultry

It is well known that the world has an urgent need for access to new effective treatments for bacterial infections in order to replace the miracle drugs or “antibiotics” of the last century [23]. Phage therapy has been considered a promising tool in eliminating bacterial infections in poultry. Research is ongoing to reduce on-farm pathogen occurrence in broilers for *Salmonella* [25,26,29,51,52,53], *Campylobacter* [54,55,56,57,58], *Escherichia coli* [53,59,60,61] or *Clostridium perfringens* [62,63,64], and in laying hens for *Salmonella* [65,66,67]. Other on-farm interventions included the control of bacteria in drinking water, shavings and on plastic surfaces [68]. Moreover, studies on post-harvest interventions using bacteriophages were conducted to control pathogen contamination in raw chicken meat against *Salmonella* [69,70] and *Clostridium perfringens* [71,72] in raw turkey meat [73] and eggs against *Salmonella* [74,75].

Nevertheless, one of the major limiting factors for the widespread use of phages is the regulatory framework of bacteriophage products [76]. Since 2011, phages have been categorized as drugs in the United States and as medicinal products in the European Union [77,78,79]. Thus, the Food and Drug Administration (FDA) and the European Medicines Agency (EMA) are responsible for their marketing and manufacturing authorization in the United States and European Union, respectively [79]. In the United States, the FDA has granted bacteriophage-based products GRAS (Generally Recognized As Safe) approval, and countries such as Switzerland, Israel, Canada, Australia, New Zealand, China and Brazil have approved bacteriophage-based products for application in foodstuffs [45,76,80]. However, there is currently no established regulatory pathway to register a phage-based product. There is a lack of consensus on how it should be regulated, regardless of whether they are intended for use as a feed additive, in pre-harvest intervention or in post-harvest application [76]. The lack of legal consensus and clarity slows the development of commercially available bacteriophage products in Europe. Although countries like Georgia, Russia and Poland have been using phages to treat infections since their discovery, there are no regulatory guidelines that can be readily adopted [78,81]. In fact, in Poland, phage therapy is categorized as an “experimental treatment” according to the *Polish Law Gazette*, 2011, item 1634 and article 37 of the Declaration of Helsinki [78]. Nevertheless, on a positive note, based on Regulation 2019/6, in 2022, the EMA published the conceptual paper on the quality, safety and efficacy of phage products as veterinary medicines in Europe. The document set out the starting point for developing the guidelines for the approval of phage-based products, and the key challenges were outlined. Among its main conclusions was the establishment of a task force for novel therapies. In 2023, the first concept paper on the quality, safety and efficacy of bacteriophages as veterinary medicines (EMA/CVMP/NTWP/32862/2022) was published, which laid down the applicable requirements for phage-based veterinary products. Moreover, in the European Union, the EFSA deemed the application of phages against *Listeria* as safe [82]. In this sense, EFSA positively evaluated the safety and efficacy of Listex™ P100 for food safety applications on different ready-to-eat (RTE) food products [80,82]. For poultry, Żbikowska [39] reviewed the different commercially available phage products, both for pre-harvest steps, such as Bafasal^®^ (Proteon Pharmaceutical, Piotrkowska, Poland), Biotector^®^ S (CJBio, Seoul, Korea), SalmoFREE^®^ (Nofima, Tromsø, Noruega), Ecolicide PX™ (Intralitix, Columbia, MD, USA), ListShieldTM Listex™ P100 (PhageGuard) (Intralytix, Columbia, MD, USA), and for the post-harvest step, such as SalmoFresh™ (Intralytix, Baltimore, MD, USA), SalmoPro^®^ (Phagelux, Shanghai, China), Salmonelex™ (PhageGuard) (Micreos, Wageningen, The Netherland), PhageGuard S^TM^ (Micreos, Wageningen, The Netherland), BacWash^TM^ (Elanco, Greenfield, MA, USA) and EcoShield^TM^ (Intralytix, Baltimore, MD, USA). 

However, there is an important factor in *Salmonella* epidemiology: the environmental persistence of the bacteria. Because of its ubiquitous nature, *Salmonella* may cycle through a flock of broilers or layers into the livestock facilities environment and back into another flock of broilers or layers [83]. In this regard, as pointed out by the EFSA and ECDC, it was observed that one out of every two *Salmonella* isolates from broilers belonged to the Infantis serovar [84], constituting up to 90% of all *Salmonella* isolates in broilers at slaughter [85]. Numerous researchers are studying the rise of this serovar in an effort to tackle the virulence factors that make it so persistent and how to effectively address it. Notably, *S.* Infantis has been found to present the pESI-like mega-plasmid (the plasmid of emerging *S.* Infantis) [11,86]. The pESI-like plasmid has been linked with superior biofilm formation, adhesion and invasion into avian and mammalian host cells. This plasmid increased *S.* Infantis fitness and antibiotic resistance, acquisition and transmission of AMR and resistance to quaternary ammonium compounds and heavy metals under various environmental conditions [11,86,87,88,89,90]. Hence, the elimination of *S*. Infantis from farms or slaughterhouses remains a current challenge, even with thorough cleaning and disinfection [86,91]. *Salmonella* persistence highlights the importance of developing novel biocontrol agents, such as bacteriophages, to combat these continuously evolving organisms. Bacteriophage-based products can serve as biosanitizers in hatcheries, farms, transport crates, poultry processing plants and food contact surfaces [39]. For example, a phage-based surface disinfectant against *Salmonella* has been marketed by the US company OmniLytics Inc. (Sandy, UT, USA) [39]. Researchers are highlighting the role of bacteriophages as a complement to the cleaning and disinfection processes across all facilities involved in the production of food, ranging from incineration and disinfection of equipment to the sanitation of the food products themselves.

## 4. Current Characteristics of Cleaning and Disinfection Used in Poultry Production and the Main Challenges: Biofilms

Good internal and external biosecurity measures are key components to prevent the introduction or reduce the infection pressure of *Salmonella* and *Campylobacter* on the farm. Hygiene measures (cleaning and disinfection) play a significant role in the epidemiology of both bacteria on two levels. Persistent environmental contamination is a major factor in the reinfection of poultry flocks [23]. Firstly, inadequate equipment maintenance, surfaces and unhygienic factory design can create niches where bacteria could find nutrients, water and protection from cleaning, allowing their survival and growth. Secondly, surface sanitization in poultry facilities and food industries can contribute to the emergence of antimicrobial resistance indirectly, mainly due to the existence of cross-resistance between biocides and antibiotics [92,93].

Current best practices for eliminating the growth of resistant bacteria require on-site treatment, including good hygiene practices and different antimicrobial agents. It is widely acknowledged that the cleaning process alone is responsible for the removal of around 90% of bacteria, and subsequent disinfection eliminates an additional 6–7% [94,95]. The presence of organic material in the environment enhances the survival of microorganisms, so its comprehensive removal is an essential step in the cleaning and disinfection process, due to its influence on the effectiveness of disinfectants. These practices must be applied at all stages of the food production process to guarantee safety and quality. When commonly used disinfectants are applied correctly, they could inhibit the colonization of introduced bacteria [96]. However, failures in disinfectant dosing and/or application to wet surfaces can lead to inadequate equipment disinfection and bacteria exposure to subinhibitory chemical levels [96,97,98]. Although desiccation processes have been shown to enhance the effectiveness of disinfection procedures [96,99], managing them can be complex when continuous or even daily production runs are required [96]. Notably, bacteria could develop resistance, when an increase in concentration or time becomes necessary in order to exert the same reduction, or tolerance, when the bacteria’s susceptibility changes due to its exposure to subinhibitory levels [96].

Disinfectants are chemicals used to remove pathogenic microorganisms and other infectious agents from surfaces and equipment. Disinfectants can have different modes of action, but in general, they act by damaging the cell membrane of microorganisms or disrupting their metabolic processes [39]. Among the various commercially used disinfectants is chlorine. Although the disinfectant properties of chlorine have been recognized since the latter half of the nineteenth century, it was not until 1988 that chlorine-containing compounds were also found to possess oxidative properties. Various chlorine-containing compounds are employed as biocides, often chosen for their combination of affordability, ease of use and high efficacy. Upon addition to water, chlorine reacts with the hydrogen and oxygen in water molecules, leading to the formation of hydrochloric acid (HCl) and hypochlorous acid (HOCl). HOCl further undergoes dissociation to generate hypochlorite (OCl^−^) and hydrogen (H^+^) ions. Both HOCl and OCl^−^ constitute the “free chlorine” in a solution and are the primary compounds responsible for the antimicrobial action of chlorine supplementation [100,101,102,103,104]. Slow-release chlorine dioxide (SRCD) is frequently applied in poultry processing to reduce *Salmonella* levels in carcasses. The necessity for utilizing SRCD as an antimicrobial stems from the reality that, despite meticulous endeavors to minimize the prevalence of live birds carrying *Salmonella*, the mechanical actions of plucking machines and unintentional damage to entrails during the evisceration stage inevitably contribute to the dissemination of *Salmonella*. In addition, the contamination levels of carcasses that end up in the supermarket are strongly correlated with the amount of cross-contamination that occurs during processing. While these chemicals have shown good efficacy, they are not allowed in the EU due to potential hazards associated with their chemical action [105,106,107].

Organic acids are a widely chosen antimicrobial option due to their high efficacy, affordability and ease of application, leading to their extensive utilization for various purposes. Organic acids inhibit bacterial growth by reducing the pH. Fundamentally, organic acids hinder the bacterial cell by inducing an accumulation of anions in the bacterial cytoplasm, adversely impacting the proton motive force (PMF) and, consequently, the cell’s capacity to sustain an optimal pH. As a result, this perturbation modifies the internal environment of the cell, hindering DNA synthesis, normal enzyme activity and cell reproduction. Multiple studies have been conducted regarding which organic acids are the most efficient in their action, resulting in significance [108,109,110,111,112,113,114,115]. A solution of lactic acid (1%) resulted in a 66% reduction in CFU/g, whereas acetic acid (1%) and citric acid (1%) demonstrated reductions of 55% and 51%, respectively. Fumaric acid is a technically effective option, but it affects the sensory properties of chicken breast more. Other options tested include succinic acid, also with good results. These studies emphasize the importance of employing organic acids in combination with other methods, such as refrigeration and freezing, to ensure a satisfactory level of reduction [108,109,110,111,112,113,114,115]. Furthermore, the antimicrobial effectiveness of organic acids is significantly influenced by factors such as contact time, temperature, acid concentration, or its combination with other agents. This complexity poses challenges, particularly in the context of the high prevalence of *Salmonella* resistance and the ongoing necessity to guarantee effective pathogen elimination through specific application methods [116,117]. While organic acids often exhibit relatively high efficacy, there is a potential risk associated with incorporating them at certain levels and temperatures, as this may impact the sensory properties of the meat [116,118,119,120].

Regardless of the many preventative measures in place, bacteria tend to live attached to surfaces and to create a complex structure known as biofilm [121,122,123]. In this sense, bacteria living under the biofilm state present a different phenotype from their planktonic counterparts [124,125] and demonstrate a high tolerance to antimicrobials, desiccation and heat [124,126]. Biofilms are complex three-dimensional structures that protect microbial communities from biotic and abiotic factors produced by the microorganisms themselves. The extracellular matrix is composed of a mixture of various polymeric compounds, such as polysaccharides, proteins, nucleic acids and lipids [127]. As in ancient Chinese warfare, where soldiers often formed an organized formation in a fortified circle to protect themselves and attack their opponents, bacteria build a protective system that is hard to achieve for individual bacteria [128]. It serves the purpose of maintaining bacteria in close proximity to one another while also establishing channels to distribute water, nutrients, oxygen, enzymes and cell debris [127]. They have inherent resistance to antimicrobial agents and are challenging to remove or eradicate with the application of disinfectants [129]. Their multilayered structure acts as a barrier and limits the diffusion of the antimicrobial agent [129]. Moreover, it includes microorganisms at various stages of metabolic dormancy in cells with different susceptibility, which favors the acquisition of new genetic traits of resistance [129]. Biofilm formation is especially relevant in *Salmonella* and *Campylobacter* epidemiology, thus also in poultry production. Approximately half of the *Salmonella* strains isolated in poultry farms present the ability to generate biofilms in poultry farm processing areas and on contact surfaces [130,131]. *Salmonella* biofilm production is mainly related to the presence of sgD, adrA and gcpA genes, which are responsible for the production of cellulose and curli fimbriae [130], while *Campylobacter* could develop monoculture and mixed-culture biofilms. Indeed, oxidative stress has been linked not only with the transformation to viable but not cultivable forms but also with biofilm formation to favor *Campylobacter* survival [132]. These bacteria are resistant to most traditional antimicrobial strategies, making it necessary to design and implement novel biocontrol agents.

## 5. Biofilms and Bacteriophages

**Figure 2:**
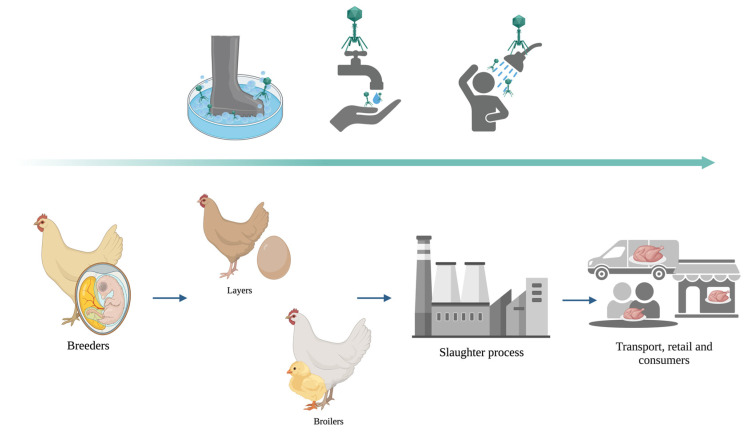
Schematic diagram illustrating phage biocontrol measures “from farm to fork”.

Effective intervention on the farm will likely require a multifaceted approach, and in the light of bacterial resistance to cleaning and disinfection protocols, it is unlikely that there will be any single “silver bullet” approach to eliminate *Campylobacter* and *Salmonella* in poultry processing. Subsequently, a prophylactic phage-based biocontrol/disinfectant agent will ensure the safety level throughout the farm-to-fork process (Figure 2).

Phages are described as nontoxic, natural agents, able to withstand extreme physiological conditions while also maintaining a narrow spectrum of antimicrobial activity [24]. Phages exert their antibacterial activity through the action of depolymerase and lysin enzymes. Depolymerases are responsible for breaking down capsular polysaccharides, while lysins target the peptidoglycan in bacterial cells [128,133,134]. Despite biofilms providing resistance to phages due to the impermeability of the biofilm matrix, phages exhibit a different mode of action on bacteria within biofilms compared to antibiotics or biocides [135]. Phages have the ability to destroy bacterial hosts, thereby preventing biofilm formation [135,136]. They can also infiltrate existing biofilms, eliminating the biofilm structure with or without destroying the resident bacteria [135,136]. The removal of biofilms using phages in nature can be classified as intra- to extracellular degradation of the bacterial structure, extra- to intracellular degradation of the bacterial structure and chemical dispersion of the biofilm matrix [135,137]. Phage-based treatment acts through three corresponding modes: basic phage therapy, phage-derived lysins and phage-derived depolymerases [133,134,138].

As mentioned above, phages can carry and express depolymerizing enzymes [135]. Depolymerases perform various activities, such as degrading EPS or cleaving structural polysaccharides like LPS or the PG glycan chain [139]. In this sense, to target biofilms, phages infiltrate biofilm using depolymerases, which are enzymes specialized in hydrolyzing the polysaccharides and polysaccharide derivatives found in the biofilm’s outer membrane [24]. Usually, this enzyme is encoded at the tail structure of the phage, a structure involved in the phage infection, thus giving phage a notable advantage over other antimicrobial agents [24]. 

The intra- to extracellular degradation of the host cell is characteristic of lytic phages [43,128], when phages induce the release of progeny phages from the bacteria host at the final stage cycle [43,128]. Throughout bacteriophage replication, the growing population of infectious progeny phages in the biofilm eliminates the bacteria responsible for the production of extracellular polymeric substance (EPS), which composes the biofilm matrix [135]. This process gradually removes the biofilm and reduces its capacity for regeneration. In fact, phages could coevolve with the bacterial biofilms, and consequently, their ability to infect adherent bacterial populations is to be expected [135]. 

Another interesting feature is that phages can induce the expression of these enzymes in their host bacteria [139]. Phages could also infect persistent cells and remain within them until reactivation occurs, which then destroys the cells [135].

## 6. The Role of on-Farm Use of Bacteriophages as Disinfectants

### Bacteriophages as Disinfectants in Poultry Pre-Harvest Stages

The phage anti-biofilm activity in an experimental poultry model has been explored. At pre-slaughter stages, phages could serve as biosanitizers in hatcheries, farms and transport crates [39]. During these phases, contamination and the dissemination of *Salmonella* are of particular relevance. Due to its epidemiology and biofilm formation, it is not only transmitted vertically but also persists in the environment, spreading horizontally and among successive flocks. Indeed, *Salmonella* persists in the litter, dried feces and feed within empty poultry houses for up to two years [140]. Furthermore, following cleaning and disinfection procedures, its survival is notably significant in drinker and feeder lines, the anteroom (including electrical panels, floors, and surfaces), farmers’ boots and fomites in the lysogenic form, the bacterium [141]. Furthermore, in caged layer houses, an additional challenge arises due to the difficulty of cleaning cage tiers [95]. However, the exterior of persistently infected houses remained substantially contaminated [141], and this fact is not usually the focus of control studies or legislation. Indeed, the surrounding farm environment sustains *Salmonella* circulation with wildlife vectors, including rodents [95,142] and insects, including flies and mealworms [143], highlighting its significant role in transmitting *Salmonella* between successive flocks. Hence, it is necessary to focus on the elimination of persistent environmental *Salmonella* from facilities, materials and the surrounding farm environment [11]. 

For example, Korzeniowski [144] studied the phage’s ability to eradicate *Salmonella* from poultry drinkers (the main point in pathogen horizontal transmission within a poultry flock) and on a stainless-steel surface. They showed that the biofilm formed by *S*. Enteritidis was eradicated from poultry drinkers and was reduced in the range of 60–97% on a stainless-steel surface. Moreover, *Salmonella* phages isolated from a chicken farm and slaughter plant demonstrated a reduction in developing and mature biofilms in 96-well microplates [145]. Similarly, a phage isolated from poultry fecal samples reduced the concentration of *S*. Enteritidis excreted by chicks through feces and in their surrounding environment and from metal surfaces [146]. Gong [147] evaluated the application of bacteriophages to reduce *Salmonella* contamination on workers’ boots in both the laboratory and the rendering-processing environments. They combined and compared phage application (cocktail), disinfection with sodium hypochlorite (400 ppm) and 30 s brush scrubbing to assess the efficacy of phages for reducing *Salmonella* on workers’ boots [147]. Under laboratory conditions, the phage treatment of *Salmonella* biofilms on boot soles resulted in a reduction of 91.5% CFU/boot when applied alone, and 97.0% and 99.2% CFU/boot when combined with hypochlorite or brushing, respectively. In a rendering-processing plant, the phage treatment of *Salmonella* biofilms on workers’ boots resulted in a reduction of 82.2% CFU/boot when applied alone, and 92.9% and 93.2% CFU/boot when combined with hypochlorite or brushing, respectively. Although the prevalence of *Campylobacter* in poultry flocks has been associated with several factors, including inadequate disinfection between chick placements and the presence of rodents and insects [148], studies on phages as disinfectants against *Campylobacter* have primarily focused only on post-harvest stages. 

## 7. Bacteriophages as Disinfectants in Poultry Post-Harvest Stages

At post-harvest stages, phages could serve as biosanitizers in poultry processing plants and on food contact surfaces. During these phases, contamination of *Salmonella* and *Campylobacter* are relevant. For both bacteria, just a few infected chickens at the slaughterhouse can contaminate the entire processing line [23]. Throughout the slaughter process, carcasses may become contaminated by bacteria present in the intestinal content of the animals, either from the same flock or in previously slaughtered flocks [21,149]. Cross-contamination of broiler meat can also take place in the consumer´s home. This is a particularly critical risk stage for *Campylobacter*. When the meat is stored under refrigeration conditions, it allows bacteria to infect other meats stored in the same packaging [21]. In this sense, *Campylobacter* can survive for up to 18 days under standard refrigeration temperatures of 4 °C without showing any decrease in bacterial counts [21,150,151,152]. On the other hand, the risk of *Salmonella* contamination in eggs extends to the post-collection, storage, transport and food handling stages [153]. Consequently, post-harvest practices are focused on the use of physical and chemical approaches, which may not succeed in reducing pathogen loads for several reasons, such as application-dependent and bacterial-resistance-related factors [76].

Phage treatment can be used to inactivate *Salmonella* and *Campylobacter* attached to food contact surfaces or grown as biofilms. In this sense, de Ornellas [154] highlighted the relation to the contact surface and the contaminant pathogen, as both are capable of interfering in the bacteria’s persistence in industrial environments through biofilm production and the ability of phages isolated from hospital wastewater and poultry wastewater to reduce *Salmonella* biofilm producer. The effectiveness of different phages in removing *Campylobacter*, *Listeria* and *E. coli* O157:H7 from the surfaces of stainless-steel, polypropylene and ceramic materials has been evaluated [127,155,156,157]. Its use is promising, although very challenging due to the diversity of bacteria found in different environments [127,155,156,157]. Siringan [158] first studied the effect of bacteriophage treatments of *Campylobacter* biofilms on glass as a matrix and demonstrated that these bacteriophages can reduce the numbers of viable bacteria and disperse the matrix. After that, Siringan [158] highlighted that in phage treatment, despite an equilibrium between host and phage, bacteria can act as expendable vehicles for the delivery of phages to new host bacteria within pre-colonized chickens. Hence, the application of phages is not expected to replace the use of disinfectants, but under particular circumstances, it could act as a complement [129,159].

## 8. Main Limitations to the Use of Bacteriophages

Despite its numerous advantages, the use of phage therapy is substantially limited due to different causes. A summary of all the information gathered in this review is presented in the following table (Table 1).

### 8.1. Limitation Due to the Phage of Choice

Individual bacteriophages cannot be used to fight broad-spectrum infections. In many cases, complex identification and characterization of the etiological agent is necessary. Another adverse phenomenon in phage therapy is that phages can be eliminated by the reticuloendothelial system, reducing their half-life in the body and limiting the effectiveness of the treatment [34,160,161].

### 8.2. Lysogenous Forms

In the lysogenic state, the bacterium gains immunity against superinfection by phages of the same type, which is an unfavorable outcome for the goals of phage therapy. Consequently, lysogenic phages are not employed in therapeutic applications [162]. An additional drawback is its capacity to transduce segments of the bacterial genome post-infection, potentially leading to the dissemination of detrimental or virulent genes within the bacterial population. The analysis of phage genomes might be a time-consuming technique, posing challenges in urgent infection treatments. Due to the heightened specificity of phage infection to a particular bacterial strain, employing multiple phages often proves more effective in managing an infection [48,163,164].

### 8.3. Legal Limitations

The regulatory approval for phage utilization lacks global standardization, exhibiting variations across different countries. In the United States, Canada, Switzerland, New Zealand, Australia and Israel, the application of phages as a processing aid is permitted. However, within the European Union, this practice is exclusively allowed in the Netherlands and is not listed in the qualified presumption of safety (QPS) list [120,165,166,167,168].

### 8.4. Need for Purification and Stabilization

Only complete characterization and screening of phages can remove those that encode toxic proteins or proteins that allow the behavior of temperate (integrative) phages. To achieve an adequate level of purification for animal model studies, ultracentrifugation using a CsCl gradient is employed, followed by the removal of endotoxins. Phage purification can also be accomplished through chromatography methods [34,169,170]. When chromatography is used for purification, endotoxin levels decrease by 10 to 30 times compared to the traditional method, albeit often resulting in a lower final phage titer. The stability of phage preparations is crucial for effective delivery over time. However, due to the unique sensitivity of each phage to chemical and environmental factors, a universal preparation strategy is not currently feasible. Typically, phages are resuspended in simple aqueous solutions. Nevertheless, prolonged storage of phage solutions may lead to a gradual loss of phage activity, necessitating the addition of stabilizers. An alternative approach involves freeze-drying phage solutions, converting them into a stable powder with a high degree of stability [48,171].

### 8.5. Dosage

One of the main obstacles to the removal of bacteria from poultry is that a significant number of phages are needed to adsorb individual host cells. The application of phages at lower doses did not provide statistically significant protection. In addition, preventive treatment in phage therapy did not prevent colonization. In many cases, the efficacy of phage therapy should be maximized by using a high titer of bacteriophages to reduce colonization [56,60,172]. An additional hurdle in the use of phage therapy is that colonization of the chicken caecum by *S.* Enteritidis and Typhimurium is inhibited for only 24 to 48 h after phage treatment, so it seems necessary to determine the optimal timing and delivery of bacteriophages in a real-life poultry industry setting [34,173]. The presented data illustrate that only elevated phage concentrations prove effective in achieving a notable reduction in both mortality rates and foodborne pathogens. Conversely, administering high doses over extended periods can lead to the development of neutralizing antibodies [174,175]. It is imperative that each phage preparation designed for application in poultry veterinary medicine, poultry production and the poultry industry be, above all, safe and efficacious. Critical considerations include the dosage, route of administration (including the preparation of standardized formulations), timing of phage-based product administration and the concurrent use of other preparations (e.g., competitive exclusion) or vaccines [39].

### 8.6. Terms of Use

The endurance of bacteriophages on or within food is subject to variability among different bacteriophages and is influenced by application conditions (e.g., dosage) and environmental factors (e.g., temperature). Refrigeration temperatures have the potential to enhance the longevity of bacteriophages on the surfaces of meat products [39,176].

### 8.7. Resistance Mechanisms

A potential concern of phage resistance arises from increased or prolonged application of phages in the food industry. In the environment, bacteria and bacteriophages exist in a cycle of coevolution, in which hosts insensitive to phages survive or prevent phage predation by transmitting corresponding resistance mechanisms [177,178,179]. Prevention of phage infection is not the only bacterial response used. Other phage resistance mechanisms focus not on preventing phage entry but on the bacterial survival of the host once infected by phages. Bacteria are able to use different pathways of action to achieve this evolutionary survival [158,180,181,182,183,184,185,186,187,188,189]. Occasionally, when phage resistance develops, bacteria increase sensitivity to antibiotics. These results demonstrate the potential to reverse existing antibiotic resistance and potentially alleviate some of the public health problems associated with antibiotic treatment [46,190].

### 8.8. Effectiveness

The primary constraint in the application of phages is their efficacy. Numerous studies indicate an initial reduction in bacteria, yet no further reduction thereafter, underscoring that phages can diminish bacteria but may fall short of complete eradication. This limitation could stem from the incapacity of phages to reach and infiltrate bacteria after progeny, emphasizing the crucial role of adequate moisture to facilitate phage dispersion [120,191,192].

Among the factors that can influence the bactericidal effectiveness of bacteriophages in food are the following:

- Food matrix: In solid foods, it will depend on the ability of the food to adsorb the phage suspension and that it is not diluted [193]. One solution that would avoid this problem would be phage immobilization. In plant compounds, substances such as organic acids and tannins would inactivate bacteriophages [193,194,195].
- pH: Given their nature, they do not seem useful when the food has a pH not between 5 and 8, extending this range to between 4 and 10 when the temperature is low [196,197].
- Temperature: Phages have a lower effect at ambient temperatures (20 °C) than at refrigeration temperatures (4 °C) [175,198].
- Multiplicity of infection (MOI): The greater the MOI, the greater the bacterial lysis, which usually requires high concentrations of phages [199,200].
- Phagoresistance: Bacteria have been described that exhibit resistance to lytic bacteriophages due to mutations [36,201].
- Combination with other control measures: Although phages are a good alternative for biocontrol, they do not usually achieve a complete elimination of the pathogen, so it is always recommended that they be applied together with other measures [202].

## 9. Key Insights into Bacteriophage Application in Poultry Farming

### 9.1. Pathogen Threat

*Salmonella* and *Campylobacter* are significant zoonotic pathogens that continue to be a major public health threat, primarily through the consumption of poultry meat and eggs. These bacteria have been responsible for numerous foodborne outbreaks. The growing concern is their antibiotic resistance, which results in more severe illnesses, treatment failures and increased hospitalizations.

### 9.2. Emerging Phage Technology

Bacteriophages, often referred to as phages, are emerging as a promising “green” technology to enhance food safety and combat persistent *Salmonella* and *Campylobacter* infections. They offer potential solutions to reduce these pathogens in poultry, both in on-farm and post-harvest applications. However, the regulatory framework for bacteriophage products is still evolving, posing a challenge to their widespread use in the food industry.

### 9.3. Persistent Bacteria and Phage Solutions

The persistence of zoonotic bacteria in the environment remains a significant challenge despite rigorous cleaning and disinfection efforts. The presence of certain plasmids can enhance their resistance and virulence. Bacteriophages are considered potential biocontrol agents to combat the persistence of *Salmonella* at various stages of poultry production, from hatcheries to farms and processing plants. These phage-based products can complement existing tools for maintaining food safety in the poultry industry.

### 9.4. Biofilm Challenge

The formation of biofilms by these zoonotic bacteria is a major obstacle in poultry production. Biofilms are complex three-dimensional structures that shield bacterial communities, making them highly resistant to antimicrobials and creating a barrier that hinders the effectiveness of disinfectants. Biofilm formation is particularly relevant in the epidemiology of *Salmonella* and *Campylobacter* in poultry production, emphasizing the need for innovative biocontrol agents to combat these persistent pathogens.

### 9.5. Multifaceted Phage Approach

Addressing the challenge of *Campylobacter* and *Salmonella* in poultry processing will likely require a multifaceted strategy, as there is no single “silver bullet” solution. Given bacterial resistance to traditional cleaning and disinfection methods, a preventive phage-based biocontrol/disinfectant approach is proposed to ensure safety from farm to fork. Phages, which are natural and nontoxic agents, are effective tools against these bacteria. They can withstand extreme conditions and have a narrow antimicrobial spectrum. Phages act through depolymerase and lysin enzymes, breaking down bacterial structures. Importantly, they have distinct modes of action on biofilms compared to antibiotics. They can prevent biofilm formation, infiltrate existing biofilms and disrupt their structure. The phage-based treatment includes three modes: basic phage therapy, phage-derived lysins and phage-derived depolymerases. These approaches offer the potential to target and eliminate biofilms, providing a valuable tool in combating *Salmonella* and *Campylobacter* in poultry processing. Moreover, phages can coevolve with bacterial biofilms and infect persistent cells, offering an adaptable solution to the problem.

### 9.6. Effective Phage-Based Treatment

Phage-based treatments have proven effective in reducing and eradicating *Salmonella* and *Campylobacter* from various surfaces, including those in direct contact with food and biofilms. These treatments show promise in preventing cross-contamination during poultry processing, which is especially critical for *Campylobacter*. Additionally, their effectiveness in addressing the persistence of these pathogens in the poultry environment, including on farms and equipment, has been highlighted. Phages offer a promising approach to eliminating persistent environmental contamination.

### 9.7. Main Phage Limitations

The use of bacteriophages for combating bacterial infections comes with several constraints that need to be addressed. These limitations include the selection of appropriate phages, the presence of lysogenic forms, legal restrictions, the need for phage purification and stabilization, determining the correct dosage, terms of use, the development of bacterial resistance mechanisms and the overall effectiveness of phage therapy. Recognizing and overcoming these limitations is crucial to enhance the effectiveness of phage therapy in the fight against bacterial infections.

## 10. Conclusions

In summary, *Salmonella* and *Campylobacter* continue to pose significant challenges to food safety in the developed world, with antibiotic-resistant strains contributing to persistent issues. Bacteriophages (phages) emerge as a promising “green” technology, demonstrating potential applications in mitigating on-farm pathogen occurrence, particularly in the context of poultry production. While regulatory hurdles exist, recent developments suggest progress in establishing guidelines for phage-based veterinary products. Phage-based treatments exhibit efficacy in eradicating *Salmonella* biofilms, providing a complementary approach to traditional cleaning and disinfection methods, thereby addressing the complex challenges associated with persistent bacterial contamination in poultry environments.

Effective biosecurity measures, on-site treatments and innovative biocontrol agents are essential components in preventing *Salmonella* and *Campylobacter* in poultry farms. The unique modes of action of phages, particularly their ability to target and eliminate biofilms, make them a promising solution. Post-harvest, phages show potential as biosanitizers, addressing contamination risks on food contact surfaces and during meat storage. Despite facing limitations such as regulatory uncertainties and bacterial resistance concerns, addressing these constraints is crucial for the successful integration of phage therapy into poultry farming practices and broader food safety initiatives.

## Figures and Tables

**Table 1:** Summary of main limitations to the use of bacteriophages.

Limitation	Description
Phage of Choice	Individual phages are insufficient for broad-spectrum infections; complex identification is needed. Elimination by the reticuloendothelial system reduces half-life, limiting efficacy.
Lysogenic Forms	Lysogenic phages confer poor results due to acquired immunity. Transduction of bacterial genome and potential transmission of harmful genes are concerns. Using multiple phages is often more effective.
Legal Limitations	Global regulatory variation in phage utilization. Permitted as a processing aid in certain countries, limited in the EU.
Purification and Stabilization	Phage characterization is essential for toxicity removal. Purification by ultracentrifugation or chromatography. Stability is crucial but varies among phages.
Dosage	High phage concentrations are needed for bacterial removal; lower doses are ineffective. Timing and delivery are critical, with potential for induced antibodies.
Terms of Use	Bacteriophage persistence varies with type, application conditions, and environmental factors. Refrigeration enhances persistence.
Resistance Mechanisms	Increased phage application may lead to bacterial resistance mechanisms. Coevolution cycles involve various resistance strategies.
Effectiveness	Efficacy is a major limitation; initial reduction is observed, but complete eradication is challenging. Factors influencing effectiveness include food matrix, pH, temperature, MOI, phagoresistance, and combination with other measures.

## Data Availability

Not applicable.
